# The Microencapsulation of Tung Oil with a Natural Hydrocolloid Emulsifier for Extrinsic Self-Healing Applications

**DOI:** 10.3390/polym14091907

**Published:** 2022-05-07

**Authors:** Abdullah Naseer Mustapha, Maitha AlMheiri, Nujood AlShehhi, Nitul Rajput, Sachin Joshi, Ana Antunes, Mohamed AlTeneiji

**Affiliations:** Technology Innovation Institute, Masdar City, Abu Dhabi P.O. Box 9639, United Arab Emirates; maitha.almheiri@tii.ae (M.A.); nujood.alshehhi@tii.ae (N.A.); nitul.rajput@tii.ae (N.R.); sachin.joshi@tii.ae (S.J.); malteneiji@tii.ae (M.A.)

**Keywords:** self-healing, tung oil, corrosion protection, hydrocolloids, emulsifier, microencapsulation, in-situ polymerization

## Abstract

In this work, tung oil was utilised as a catalyst-free self-healing agent, and an in-situ polymerization process was applied to encapsulate the tung oil core with a poly(urea-formaldehyde) (PUF) shell. The conventional poly(ethylene-alt-maleic-anhydride) (PEMA) polymer was compared to a more naturally abundant gelatin (GEL) emulsifier to compare the microcapsules’ barrier, morphological, thermal, and chemical properties, and the crystalline nature of the shell material. GEL emulsifiers produced microcapsules with a higher payload (96.5%), yield (28.9%), and encapsulation efficiency (61.7%) compared to PEMA (90.8%, 28.6% and 52.6%, respectively). Optical and electron microscopy imaging indicated a more uniform morphology for the GEL samples. The thermal decomposition measurements indicated that GEL decomposed to a value 7% lower than that of PEMA, which was suggested to be attributed to the much thinner shell materials that the GEL samples produced. An innovative and novel focused ion beam (FIB) milling method was exerted on the GEL sample, confirming the storage and release of the active tung oil material upon rupturing. The samples with GEL conveyed a higher healing efficiency of 91%, compared to PEMA’s 63%, and the GEL samples also conveyed higher levels of corrosion resistance.

## 1. Introduction

The significant corrosion of metals through chemical reactions and structural weakening reduces their performance capability and, in some cases, renders them impracticable in certain applications. For example, in the marine industry, a disparaging process that results in significant economic losses is associated with metals’ corrosion. The replacement or repair cost of these metallic parts can result in various safety issues and additional cost. Efforts to reduce these factors include coating the metal in organic material to create a protective barrier on the metallic area. However, even so, the organic polymer coatings are still susceptible to micro and nano-level damages, such as scratches and dents, usually during the handling of the material [1]. Such damage can be challenging to identify, which could proliferate the corrosion process, rendering the coating nonbeneficial. To mitigate this issue, ‘self-healing’ (SH) polymers introduce active form of protection, in which a self-regenerating polymer with an active material can be incorporated.

SH is a process in which materials can repair/heal themselves when damaged by chemical, thermal or mechanical stimuli, allowing them to restore their original properties. SH can be categorized into intrinsic and extrinsic. Intrinsic SH concerns the material’s inherent ability to heal itself through the presence of specific reversible chemical bonds. Such bonds can include ionic interactions, hydrogen bonds, halogen bonds, radical-based systems, π—π interactions, and metal-ligand interactions [2]. In contrast, extrinsic SH concerns the set of materials that are usually sequestered from a matrix, such as a micro/nano capsule. When the host matrix is damaged, the healing agent is released into the damaged zone to conduct the healing process via a chemical reaction [3]. The SH agent is used as the material that performs the SH process, and common SH agents include natural drying oils, epoxy resins, cyanoacrylates, methyl methacrylate, hydrogel, and bacteria-based microcapsules [4].

Various examples of SH coatings can include the application of a SH agent in malleable polymers, polyelectrolyte complexes, and the encapsulation of a healing agent in nano/microcapsules [1,5,6]. Such nano/microcapsules are usually polymeric spherical structures loaded with drying oils or resins, in which a stimulus would initiate the SH process, such as mechanical damage, pH changes, temperature change, desorption, or ion exchange [1]. The application of such coatings is a relatively recent concept in corrosion protection technology and is one that seems to be extremely promising.

Drying oils can be used as oxidative SH agents and core materials in the microcapsules [1,7,8]. In this case, polymerization can occur without a catalyst, simply through the interaction with oxygen in the atmosphere. The rapid curing (cross-linking of polymer chains) of these oils is usually associated with the unsaturated conjugated systems in their structure [4]. Tung oil has been employed in various applications, such as varnishes, paints, printing inks and oil cloths, due to its ability to form a tough solid film after it polymerizes. The curing rate of the oil corresponds to the degree of unsaturation, in which faster cross-linking occurs when the degree of unsaturation is high [7]. Examples of the application of tung oil PUF microcapsules includes the work carried out by Paolini et al. [9]. Their work compared tung oil microcapsules with copaiba oil, which created average microcapsule diameters of 22 µm and 25 µm, respectively. It was also observed that the tung oil microcapsules provided more positive results for corrosion protection via open circuit potential (OCP) testing than the copaiba oil samples. Furthermore, Li et al. [10] encapsulated tung oil with a PUF shell, in which an average diameter of 105 µm was observed, and a core content of 80%. Additionally, scratch testing and immersion in NaCl solution concluded that steel plates coated with epoxy resin embedded with microcapsules exhibited improved corrosion resistance.

To prevent SH oils from curing in atmospheric open conditions unintentionally, they can be protected via microencapsulation processes. This concerns the entrapment of droplets by a protective wall material, providing a protective barrier between the core material and oxygen [11]. A microencapsulation method known as ‘one-step in-situ polymerisation’ is commonly utilised to encapsulate various core materials for multiple applications. Amino resins, such as urea-formaldehyde (UF), is an excellent shell material candidate for the encapsulation of SH core materials. The benefits of the amino resins include chemical and water resistance, long term storage capability, high mechanical strength, high loading, good thermal stability, and low permeability [12,13,14,15]. The most crucial parameters to consider are the temperature, emulsification speed, pH, reaction time, and emulsifier type and concentration [13,16,17,18,19,20].

With the one-step in-situ polymerization process, the emulsifier dramatically affects the quality of the microcapsules in terms of the morphology, size distribution, surface roughness and shell barrier properties [13,19,21]. Emulsifiers have many functions in the polymerization process, such as reducing the interfacial tension between the water and oil phase and forming micelles that can stabilize monomer droplets in an emulsion form and stabilize the growth rate of monomer/polymer species [22]. For this process, a frequently used emulsifier is poly(ethylene-alt-maleic anhydride) (PEMA), a synthetic polymeric emulsifier. For example, in a one-step in situ polymerization process, Farzi et al. [23] encapsulated cerium nitrate with a PUF shell and PEMA as the emulsifier. It was observed that the microcapsules did not exhibit uniform morphology, with approximately 92% payload. Other synthetic polymers used in this process include polyacrylamide, poly(ethyl enamine), poly(ethylene glycol), poly(methyl methacrylate, poly(acrylic acid) and poly(vinyl alcohol) [19]. Yoshizawa et al. [21] further investigated alternative compounds to PEMA that can also be used as emulsifiers, including poly(olefin-maleic anhydride) and poly(acrylic acid). They stated that the surfactant must contain a carboxyl or maleic acid group to form a polyurea microcapsule membrane, which PEMA contains.

However, hydrocolloids can be used as naturally abundant emulsifiers, which is a strong advantage in terms of availability for industrial scale-up. Additionally, hydrocolloids are more environmentally friendly and biodegradable, which reduces the overall carbon footprint of this process. Therefore, gelatin (GEL) is suggested to be used as an alternative to the very commonly used PEMA emulsifier, reducing the environmental footprint of this process. GEL is derived from natural sources and not chemically synthesized, and it is extracted from the raw collagen from animals, usually raised for consumption. Thus, the usage of GEL promotes the full use of these animals, contributing to a more ‘zero-waste’ food economy. Nonetheless, further work needs to be carried out in this field to further explore alternative environmentally viable candidates to replace synthetic polymers in the microencapsulation process. Potential feasible candidates for this process include gum Arabic, xanthan gum, pectin, chitosan, methylcellulose, guar gum and locust bean gum [19,24]. Zhang et al. [24] encapsulated a volatile phase change material (PCM) with the use of xanthan gum and methylcellulose, in which the results conveyed that the xanthan gum produced microcapsules with superior core material retention to methyl cellulose. Yu et al. [25] encapsulated thermochromic compounds with gum Arabic as the emulsifier, in which there was an immediate emulsification effect of the core, and the microcapsules maintained the thermochromic ability [25].

In the present work, the emulsifiers used are PEMA and GEL to compare a synthetic emulsifier with a naturally abundant bio-based option. Tung oil was selected as the healing agent. Currently, there has been no work carried out to encapsulate tung oil as a SH agent with the use of GEL as an emulsifier. Subsequently, after optimizing the encapsulation procedure, various characterizations of the SH microcapsules were then carried out. Such characterizations include morphology, size distribution, chemical and crystalline structures and thermal properties. Additionally, a novel and innovative FIB method is also utilised to confirm the storage and release of the active tung oil material. Additionally, the self-healing processes, and the corrosion resistance of the epoxy coating loaded with synthesized microcapsules were also showed. The aim of this study is to improve the barrier and morphological properties of micro-capsules, as well as increase the payload, yield, and encapsulation efficiency for process optimization.

## 2. Materials and Methods

### 2.1. Materials

The following chemicals were acquired from Sigma-Aldrich (Gillingham, UK): formaldehyde solution (104003, ACS reagent, about 37.0% in solution, resorcinol (398047, ACS reagent, ≥99.0%), poly(ethylene-alt-maleic-anhydride) (188050, average Mw 100,000~500,000 g mol^−1^), gelatin (04055, from porcine skin), ethanol (24102, 99.8%). Tung oil (100% pure) was bought from Hopes (Fort Mill, SC, USA). Ammonium chloride (99.5%) was acquired from Daejung, Siheung-si, Korea. Hempaprime Multi 500 epoxy primer and Hempathane curing agent 97050 were purchased from Hempel, Abu Dhabi, United Arab Emirates. All the chemicals listed were used without any additional modification.

### 2.2. Microencapsulation Process

The microencapsulation of the tung oil was carried out via one-step in-situ polymer-ization. The emulsifier solutions were pre-prepared before the experiment, by mixing 0.5 g gelatin and 0.5 g PEMA in 150 g distilled water, respectively, in a 300 mL beaker. Using a Kern ABT 100-5NM balance, 2.5 g urea, 0.25 g resorcinol, and 0.25 g ammonium chloride were measured in the pre-prepared 300 mL beaker. Using a Thermo Scientific (Waltham, MA, USA) HPS RT2 Advanced stirrer, this solution was stirred at room temperature until a clear solution was observed. The pH of the solution was adjusted to pH 3.5 (to promote the polymer condensation reaction) using a Mettler Toledo (Columbus, OH, USA) SevenCompact Duo pH meter, by adding a diluted 1 mol L^−1^ HCl solution.

The prepared solution was then placed under a Silverson L5M-A homogenizer at 2500 rpm. An amount of 10 mL of the tung oil was added dropwise into the solution, and this was left for 30 min to stabilize the oil droplets. A stainless-steel baffle was then placed in the beaker, and the solution was then placed in a LabTech LWB-111D water bath, at 25 °C, and 6.5 mL of formaldehyde was injected. Then the solution temperature was raised to 55 °C and maintained at this temperature for 4 h (the usual reaction time for successful encapsulation [26]). After the 4 h elapsed, the solution was cooled down to 25 °C. 

Subsequently, after the reaction was completed, the microcapsules were separated using a separation funnel, and washed 5 times with water (30 °C) using a vacuum filtration process. The samples were then left to dry overnight for a 12-h period, ready for storage and future use. Three batches of each sample were formulated.

### 2.3. Payload, Yield, and Encapsulation Efficiency

To analyze the payload of the of the microcapsules, dried powdered microcapsule samples were weighed, and placed in a circular compression die, to form a compressed tablet. The samples were then compressed with a Lloyd Instruments LS100 Plus Materials Testing Machine. A maximum force of 80 kN at 10 mm·min^−1^ for 240 s was used to compress the dry microcapsules to breakage, to release the tung oil. Successively, the capsules were left to dry in an oven at 150 °C for a duration of 24 h, for further drying of the compressed shell. The dried and compressed capsule shells were then weighed. The payload of the formulated microcapsules (*PL*) which is the mass ratio of the core materials to the microcapsules was calculated by [20]:

(1) $$PL=1-\frac{W_{dc}}{W_{d}}$$

where $W_{dc}$ is the weight of the compressed microcapsules, and $W_{d}$ is the weight of the uncompressed microcapsules.

The yield of the formulation process which is the mass ratio of the product to raw materials was then calculated by:

(2) $$Yield=\frac{W_{t}}{WRtot}$$

where $W_{t}$ is the total mass of the microcapsule products after the formulation process, and $W_{Rtoot}$ is the weight of all the materials used for synthesizing the shell and core, excluding the deionized water.

The encapsulation efficiency (*EE*), which is the percentage of the encapsulated core materials, was then calculated by:

(3) $$EE=1-\frac{W_{t\;}\times PL}{Tung_{in}}$$

where $Tung_{in}$ is the total amount of tung oil injected in the homogenization process.

### 2.4. Characterization process

#### 2.4.1. Microscopy of Microcapsules 

To capture the bright-field images and to observe the shape morphology of the microcapsules, an Optical Microscope DSX 1000 with a DSX10-SXLOB lens was utilized. Differential interference contrast (DIC) was also used in the OM imaging process. This technique introduces contrast to images of samples which would otherwise have hardly noticeable contrast when viewed using brightfield microscopy. Therefore, the images produced using DIC have a pseudo 3D-effect.

In addition, scanning electron microscope (SEM) imaging was carried out in a dual beam system, Scio2 (Thermo Fisher Scientific). The electron column is equipped with a Schottky field emission gun (FEG) source which gives a high resolution of <1 nm at optimized condition. The system supports advanced scanning strategies (Thermo Scientific SmartSCAN™) which allows line averaging and interlaced scanning in addition to Drift Corrected Frame Integration (DCFI). The ion column has liquid Ga ion emitter that provides focused ion beam (FIB). The ion beam can achieve a resolution of 3.0 nm. In our experiments, FIB milling process was used to cut the microcapsules. A beam energy of 30 keV and currents in the range of 1–7 nA were used during the milling process. To increase the conductivity of the samples, the microcapsules were coated with ~5 nm of Chromium with a Quorum Q150R ES sputter.

ImageJ (an image processing programme) was used for the quantification of the microcapsule shell thickness. A scale bar was set with a calibration setting, allowing for the evaluation for the shell thickness, in which a mean value was obtained.

#### 2.4.2. Particle Size Dstribution 

The microcapsule size distributions were characterized by Malvern Mastersizer 2000 particle analyser with a wet dispersion unit (Hydro 2000S). Deionized water was used as dispersant. Each experiment was carried out in quintuplets, with samples measured straight from the aqueous solution. The Malvern software computed the average size distribution, evaluating the average particle sizes, the span, and the D[3,2] (Sauter mean diameter) parameter. The D[3,2] was selected due to its sensitivity to surface area.

#### 2.4.3. Fourier Transform Infrared Spectroscopy

Attenuated total reflection (ATR) mode and Fourier transform infrared spectroscopy (FTIR) were carried out using a Bruker (Billerica, MA, USA) FT-IR Microscope (LUMOS II). The ATR–FTIR spectra were used to measure an infrared spectrum of microcapsules with a wavelength range of 800–4000 cm^−1^. The number of scans was set to 16, with a resolution of 4 cm^−1^. The samples were prepared as thin tablets by a Lloyd Instruments LS100 Plus Materials Testing Machine, with a force of 40 kN, for observation in the spectrometer. Origin Pro 2021b (a data analysis software) (Northampton, MA, USA) was then used to analyze the peaks of the spectra, with the use of the Origin Pro 2021b Gaussian peak analyzer, and the baseline anchor method selected.

#### 2.4.4. Thermogravimetric Analysis

The Thermogravimetric Analysis (TGA) was performed using a Simultaneous Thermal Analyzer (STA, Netzsch, Selb, Germany, STA449-F5 Jupiter). The TGA measured the changing of the mass over time by changing the temperature. Sample masses between 10 mg and 15 mg were loaded in an aluminium crucible with a lid and heated from 50 to 550 °C under N_2_ atmosphere at the rate of 10 °C min^−1^.

#### 2.4.5. X-ray Diffraction

The crystallographic structure for the microcapsules was tested at room temperature (25 °C) by a Bruker D8 Advance X-Ray Diffractometer with a Cu Tube (1.5418 Å) and a LYNXEYE XE-T detector. The XRD parameters were adjusted as follows, an angular increment of 0.01°, a current of 40 mA, an operating voltage of 40 kV, and a scanning rate of 0.8 s/step. The samples were prepared into a silicon ingot and collected in the range of 2θ = 10–70°.

#### 2.4.6. Preparation of the Self-Healing Epoxy Coating

The microcapsules were dispersed into the Hempel Hempaprime 500 epoxy resin primer and Hempathane curing agent 97050, and gently stirred with a glass rod mixer. Subsequently, the coatings (the pure epoxy primer and the primer mix containing the microcapsules) were sprayed onto A36 steel plates (15 × 10 × 3 cm), with a Devilbliss GTI Spray Gun (1.8 bar with a 0.5 mm nozzle). This thickness of the coatings was then verified by a GE Instruments CL5 Thickness Gauge with an average of 20 data points, with an average thickness of 177 µm.

#### 2.4.7. Corrosion Resistance and Self-Healing Performance

A scalpel was used to produce scratches on the coated samples. The width of the scratches was approximately 30 μm, and the length of each scratch was 1 cm. Corrosion resistance of the coatings was visually detected after submerging the samples in 3.5 wt.% NaCl solution for a period of 24 and 48 h, at a temperature of ~25 °C.

#### 2.4.8. Adhesion Testing

Pull-off tests were carried out to investigate the adhesive tests for the epoxy primer with the steel substrate, with and without the addition of microcapsules. Therefore, the force required to detach the test dollies glued to the primer layer from the underlaying substrate was tested The tests were carried out according to ASTM D4541-09 standards.

### 2.5. Statistical Analysis

For statistical analysis of the results, Analysis of Variance (ANOVA) and the Tukey HSD (Honest significant difference) methods were used. ANOVA is a set of statistical models and their related estimate processes (such as the “variation” among and across groups) used to assess the variances between groups’ means (or averages). The Tukey HSD test is a statistical method for determining if the relationship between sets of data is statistically significant [27]. The ANOVA and Tukey’s analysis methods were carried out with Astatsa software (2016, Navendu Vasavada). With this analysis, we can obtain the ‘*p*-value’, which is a metric that expresses the likelihood that an observed difference may have occurred by chance. The statistical significance of the observed difference increases as the *p*-value decreases. A value of (*p* ≤ 0.05) indicated a significant difference between the means. Additionally, The Q statistic is used to separate the variability seen between studies into that which is due to random fluctuation and that which is due to possible differences between tests [28].

## 3. Results and Discussion

### 3.1. Gelatin as an Alternative Emulsifier

In the production of PUF microcapsules, PEMA is a particularly popular emulsifier in the one-step in situ polymerization process. There have been various examples of researchers utilising this emulsifier for various applications. For example, Brown et al., [26] produced PUF microcapsules with a dicyclopentadiene core material, to be used for self-healing applications. Furthermore, Fan and Zhou [29] studied the effect of emulsifiers on the encapsulation of tetrachloroethylene, in which they suggested that PEMA produced very rough microcapsules with a large size distribution and a mean diameter of 108 µm.

**Figure 1:**
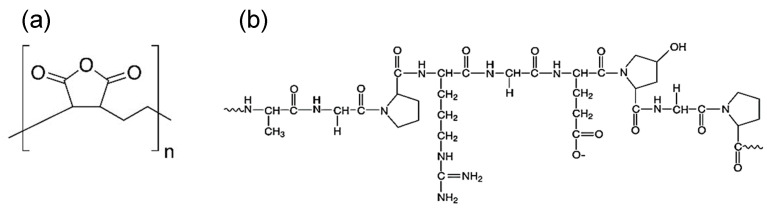
Chemical structures for (**a**) PEMA and (**b**) GEL.

GEL (from porcine skin) is then proposed to be employed in the emulsification process as a more contemporary and naturally abundant option. The chemical structures of the PEMA and GEL compounds are shown in Figure 1. As seen in in Figure 1a, PEMA contains anhydride groups, which when hydrolysed, convert into carboxyl (COOH) groups [21]. Generally, carboxyl groups are suggested to accelerate the polycondensation reaction in urea-formaldehyde, in which high molecular weight polymers are produced, leading to much rougher shells.

GEL contains other functional groups that may partake in the polycondensation reaction, including hydroxyls and amines, as seen in in Figure 1b. Previously, Yoshizawa et al. [21] claimed that carboxyl or anhydride groups are a necessity in producing PUF microcapsules, but this may not necessarily be the case, as alternative functional groups such as hydroxyl and amino groups may also partake in the polycondensation process.

**Figure 2:**
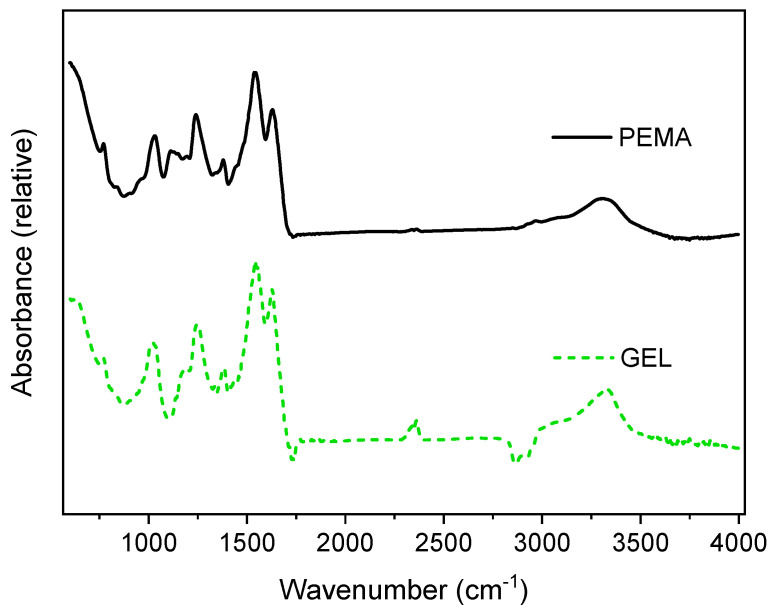
FTIR analysis for the PUF resin produced with PEMA and GEL emulsifiers.

FTIR analysis was carried out for cured PUF resins produced with the aid of the PEMA and GEL emulsifiers to compare the chemical composition of the two samples, as shown in Figure 2. Both samples exhibited peaks at 1035 cm^−1^ and 1130 cm^−1^, which are assigned to methylene bridges (–NCH_2_N–), and C–O aliphatic ether, respectively. The peak of 1240 cm^−1^ is attributed to the stretching of C–N and N–H of tertiary amines. The peak at 1372 cm^−1^ for both samples was assigned to the –CH_2_OH, illustrating the typical reaction between urea and formaldehyde [24]. Furthermore, the peaks at 1150 cm^−1^ and 1630 cm^−1^ were attributed to the C–N stretching of secondary amines and the stretching of carbonyl groups, respectively. Both samples also conveyed a broad peak at 3200–3400 cm^−1^, which is usually assigned to the N–H stretching attributed to secondary amines. These results agree with other FTIR measurements of cured PUF resins [15,30]. Additionally, the occurrences of the bands confirmed the polymerization of PUF resins during a curing process. This spectra is in agreement to many studies in the formulation of PUF resins [14,15,30,31,32].

### 3.2. Formulation of Microcapsules with PEMA and GEL Emulsifiers

**Figure 3:**
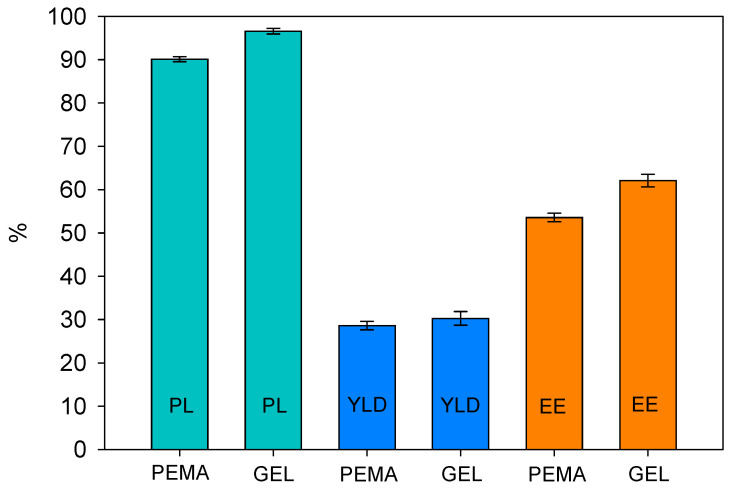
The payload (PL), yield (YLD) and encapsulation efficiency (EE) for the microcapsules formulated with PEMA and GEL as emulsifiers.

Microcapsules were then formulated in triplicate, with 0.3 wt.% PEMA and GEL to study the mean values for initial payload, yield, and encapsulation efficiency, which can be seen in Figure 3. From the initial results, GEL had higher values in all three categories. For example, GEL exhibited payload values of 96.5%, while PEMA had 90.8%, conveying that the GEL capsules contain significantly more active tung oil. Furthermore, according to Tukey’s HSD analysis in Table 1, there was a significant difference in the mean values between the PEMA PL and GEL PL. In comparison, Li et al. [10] formulated tung oil microcapsules with PEMA as the emulsifier, garnering a payload of 80%, while Brown et al. [26] employed PEMA to encapsulate dicyclopentadiene (DCPD), resulting in a payload of 87.5%.

Furthermore, both batches produced similar yields, with PEMA and GEL producing 28.6% and 28.9%, respectively, which is not deemed to be a significant difference, also confirmed by Tukey’s analysis. In terms of EE, the PEMA batches resulted in a value of 52.6%, and the GEL samples produced 61.7%, which is the most significantly improved parameter, and Tukey’s analysis conveys a significant variation in the mean values of the results also. Overall, this preliminary indication of results conveys the initial benefits of utilising the GEL microcapsules in process efficiency and active core material encapsulation. For industrial applications, factors such as payload, yield and encapsulation efficiency are important in terms of efficiency and cost-saving considerations.

### 3.3. Morphology and Shell Thickness

**Figure 4:**
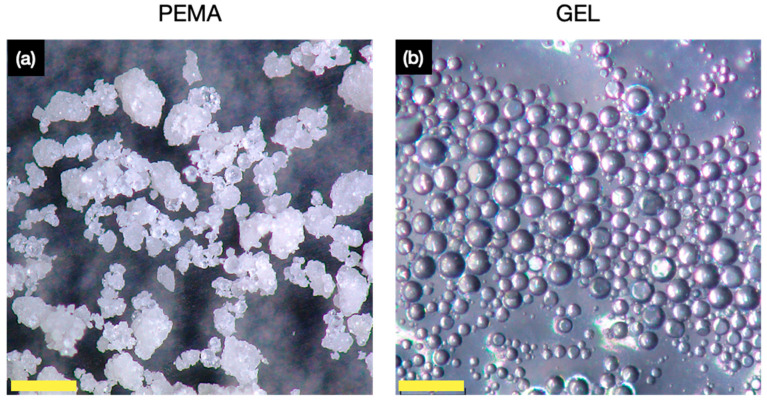
OM images for the tung oil microcapsules produced with (**a**) PEMA emulsifier, and (**b**) GEL emulsifier. All scale bars are 200 µm.

OM images were then obtained for the PEMA and GEL samples, in which a distinct variation in morphology and uniformity of the microcapsules was observed. Figure 4a conveys the microcapsules produced with PEMA, and from primary observation, it is evident that the microcapsules had a very coarse appearance with a wide size distribution. This, of course, is undesirable for controlled thickness coatings, such as for the implementation into coatings for corrosion protection, as a wide size disparity would result in an uneven layer and potential mechanical strength variation for the microcapsules. However, compared to the PEMA, the GEL samples, as shown in Figure 4b, display a much more uniform size distribution with less coarse microcapsules formulated. This represented a drastic improvement in the results and is an early indication that GEL, as a natural alternative, may yield more desirable microcapsules in terms of shell barrier properties and morphological uniformity.

**Figure 5:**
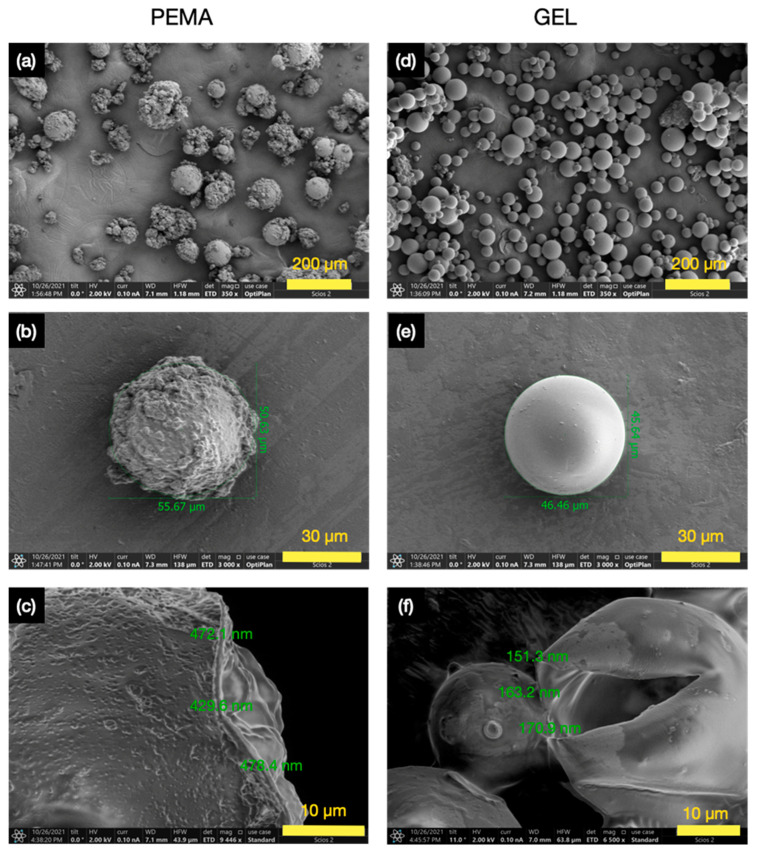
SEM micrographs for the (**a**) overall morphology for PEMA microcapsules, (**b**) individually isolated PEMA microcapsule, (**c**) shell thickness of a PEMA microcapsule, (**d**) overall morphology for GEL microcapsules, (**e**) individually isolated GEL microcapsule, (**f**) shell thickness of a GEL microcapsule.

SEM images were then taken for the PEMA and GEL samples, as shown in Figure 5. The overall PEMA samples in Figure 5a convey results in agreement with the OM images, in which very rough microcapsules were yielded. Furthermore, there also seemed to be PUF particles alongside the microcapsules, even after the filtration period, and these PUF particles would add no value to the self-healing applications. The individually isolated PEMA microcapsule, as shown in Figure 5b, more clearly conveys the rough exterior, and it seems that there are large satellite ‘PUF surface particles’ on the outer shell of the microcapsule. This morphology seems to be common with microcapsules formulated with PEMA as the emulsifier, with similar surface properties conveyed by various researchers [19,21,30,31].

The shell thickness of the PEMA microcapsule was ~460 nm, as shown in Figure 5c. In comparison, the GEL samples in Figure 5d convey much more attractive results. It is shown that there is more size uniformity, with no remaining isolated PUF particles. The individually isolated microcapsule in Figure 5e also dramatically conveys the difference in the outer shell properties compared to PEMA, bestowing a smoother shell with much fewer satellite particles. The shell thickness of the GEL samples was also much thinner than the PEMA samples, as shown in Figure 5f, with an average value of ~162 nm, approximately 65% thinner than the PEMA microcapsules.

This variation in results is suggested to be attributed to the functional groups that the emulsifiers contain. As PEMA has anhydride groups that hydrolyse in water to produce carboxyl groups, it is proposed that the carboxyl groups speed up the polycondensation reaction to produce the PUF thermoset polymers in a much more accelerated manner than the GEL emulsifier, which agrees with previous reports [19,21]. Therefore, this rapid increase in the reaction rate suggests the observed shell roughness and thickness of the PEMA microcapsules due to the large PUF particles produced in a faster reaction time. Moreover, Zhang et al. [19] also stated that when producing PUF phase change material (PCM) microcapsules, faster reactions were proposed to have taken place when emulsifiers with carboxyl groups were introduced, compared to amino or hydroxyl groups. This seems to be in agreement with the OM and SEM results, as the increased rate of reaction due to the carboxyl groups in PEMA may then lead to the larger surface particles on the microcapsules, as well as the large variation in morphology and thicker shells. A more controlled reaction rate would result in a more controlled deposition of the shell material on the microcapsule membrane, as seen in the GEL samples. A study was also carried out by Pizzi, Garcia and Wang [32], in which maleic anhydride groups were utilised to increase the rate of reaction within phenol-formaldehyde (PF) groups, in a similar fashion. As organic anhydrides hydrolyse in water, the maleic anhydride groups in the PEMA emulsifiers is also likely to hydrolyse in the reaction, in order to form hydroxyl groups, which in turn accelerates the rate of reaction in this case. This is also in agreement with the study carried out by Yoshizawa et al. [21].

### 3.4. Particle Size Distribution and Reaction Profile

**Figure 6:**
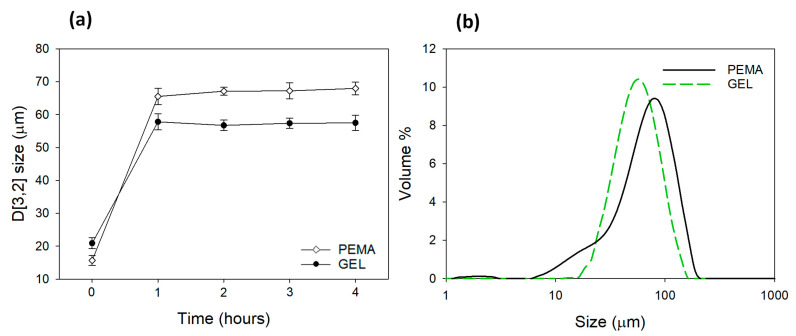
(**a**) The in situ reaction monitoring of the particle sizes (D[3,2]) over a 4 h period for microcapsules produced with PEMA and GEL emulsifiers (**b**) the final size distribution for the microcapsules formulated with PEMA and GEL emulsifier after the complete reaction.

Measurements were then carried out to observe the evolution of the tung oil droplet sizes over time and during the reaction process until the polymerization process was completed after 4 h (the duration of the reaction). This was carried out to observe the effect that the emulsifiers impose on the reaction and how the morphology of the microcapsules are influenced. As shown in Figure 6a, the microcapsules formulated with the GEL emulsifier had an average size distribution of ~21 µm prior to the polycondensation process, and after one hour, this average size increased to ~57 µm during the reaction. This may be due to the coalescence of the oil droplets until a stable droplet size is achieved. The size distribution seemed to stabilize after this time, in which an average value of ~58 µm was obtained. The microcapsules formulated with the PEMA emulsifiers had an initial droplet size of ~16 µm, and after one hour, this value rose to ~66 µm, in a more pronounced manner than GEL. The average size distribution was also less consistent with the five repeats carried out than the GEL samples at 1 h. Furthermore, at 2–4 h, the particle size was not as stable as the GEL samples and reached a final value of ~68 µm.

The final size distributions can be observed in Figure 6b, in which the PEMA microcapsules can be seen to have a wider size distribution, with an average size of ~63 µm and a span of 1.68. The GEL microcapsules resulted in an average size of ~56 µm and a span of 1.28. The homogenization speed for both processes was 2500 rpm and is the main method for controlling the overall microcapsule size. Nevertheless, as this value was used for both samples, and there is still a difference in the morphology and size distribution for both samples, it is clear the degree to which the emulsifiers affect the barrier properties of the microcapsules. The PUF particles engendered by the PEMA emulsifiers are also a contributing factor to the variation in size distribution. It is not a desirable feature when employing microcapsules for practical applications.

Zhang et al. [24] studied the evolution of PUF precipitates over time similarly using a masterziser over 2 h. The limitations of their study are that the interpretation of the results was not truly accurate due to possible agglomeration of the PUF precipitates. However, it still acts as interesting guidance to infer precipitation growth rate. Three emulsifiers were used in their process, namely xanthan gum (XG), poly(vinyl alcohol) (PVOH) and methylcellulose (MC). Interestingly, the XG and PVOH both produced larger diameter precipitates than the methylcellulose. It is speculated that the interfacial dilatational rheology of the water/oil interface could play a role in these results, as both XG and MC are used for gelling or thickening purposes. Still, the interfacial properties between the oil/water interface and the functional groups of the emulsifiers are likely to result in the variation in microcapsule properties [24].

### 3.5. Thermogravimetric Analysis

**Figure 7:**
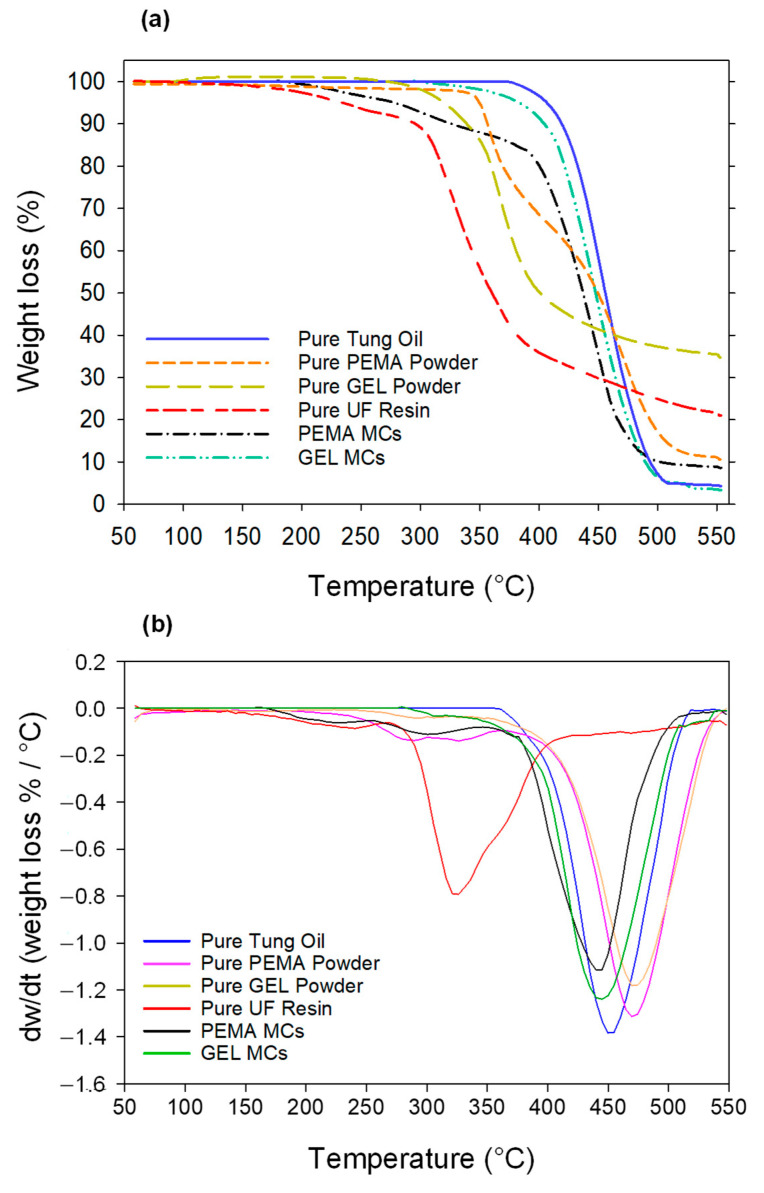
(**a**) TGA plot and (**b**) the derivative weight change for the pure tung oil, pure PEMA powder, pure Gel powder, pure UF resin, PEMA microcapsules and GEL microcapsules.

The thermal stability of the microcapsules plays an imperative role in their applications as self-healing agents. Therefore, TGA analysis was carried out to study the thermal decompositions for the pure tung oil, the pure PEMA powder, the pure GEL powder, the pure UF resin, the PEMA microcapsules, and the GEL microcapsules. The results are presented in Figure 7, alongside the derivative weight change analysis with respect to temperature. The pure tung oil initiated the decomposition process at 377 °C, indicating that the tung oil had superb thermal oxidation stability, which is in agreement with the derivative weight changes observed in Figure 7b. For the degradation pattern of the pure UF resin, there was a steady loss of mass (11% mass) from 50 to 291 °C, which is usually attributed to the gasification of small molecules such as low molecular weight polymers, formaldehyde, and water [33]. The derivative weight change clearly shows that the pure UF resin initiated the decomposition process at a much lower temperature than the rest of the samples. The majority of the mass loss was observed between 288 °C and 395 °C, and this is attributed to the UF resin undergoing pyrolysis [34]. From 395 °C to 550 °C, there was a reduced rate of mass loss, in which the total mass loss was 89%.

For the PEMA microcapsules, there was an initial mass loss of 16% up to 390 °C, and in the second degradation step between 390 °C and 497 °C, there was an additional 72% mass loss, with an overall mass loss of 91% at 550 °C. Compared to the PEMA microcapsules, the GEL capsules exhibited a thermal decomposition process at a higher temperature, and at 390 °C only 6% mass loss was observed compared to PEMA’s 16%. The majority of the GEL microcapsule mass loss was observed between 400 °C and 500 °C, in which there was an 84% reduction in mass, with a final mass loss of 96% at 550 °C. As the total mass loss for the PEMA and GEL were 91% and 96%, respectively, this also indicates the payloads of the microcapsules. Moreover, these values correspond with the payload measurements carried out earlier, with compression payload values of 89.6% and 96.2% for the PEMA and GEL, respectively. The initial decompositions of the microcapsules are usually attributed to the evaporation of free formaldehyde and adsorbed water [35]. The degradation pattern of the microcapsules follows more closely the degradation pattern of the tung oil rather than the UF polymer. However, there is a variation in the final mass loss for the PEMA and the GEL microcapsules. With the PEMA observing a total of 89% mass loss, and the GEL observing a total of 96%, this may be due to the oil/core ratio for the microcapsules and the shell thickness. As mentioned previously, the shell thickness of the PEMA microcapsules is approximately ~460 nm, while the GEL had an average shell thickness value of ~162 nm. This disparity in the shell thickness may also represent the 7% difference in the final mass loss between the PEMA and the GEL, in which the additional remaining PUF shell for the PEMA samples did not decompose to the same degree as the thinner GEL shell.

Additionally, the pure PEMA and GEL powder were studied in the same conditions. It can be observed that for the pure PEMA powder, the decomposition process was initiated at 349 °C, with a final mass loss of 89.5%, higher than that of the PEMA microcapsules. However, the pure GEL powder initiated the decomposition process slightly earlier than the PEMA powder at a value of 315 °C, but nonetheless, the final mass loss for the GEL is 65.3%. The GEL powder showed the least amount of mass loss for the samples overall. Although the pure GEL powder exhibited higher thermal stability than the pure PEMA powder, it is still evident again that perhaps the shell thickness of the PEMA microcapsules results in a lower mass loss than the GEL microcapsules ultimately.

### 3.6. XRD Investigation

**Figure 8:**
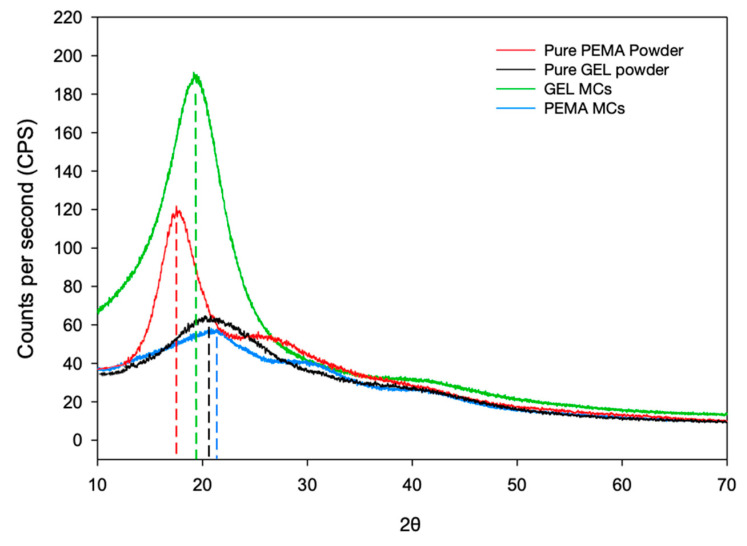
XRD scans for pure PEMA powder, the pure GEL powder, and the microcapsules produced with PEMA and GEL as the emulsifier.

XRD analysis was performed to study the crystalline nature of the shell materials produced by employing PEMA and GEL as emulsifiers, and the pure PEMA and GEL powders. This is shown in Figure 8. The pure PEMA powder had a higher CPS peak value than the pure GEL powder, with values of 118 at 17.4° and 65 at 20.5° respectively. In terms of the shell material produced with the PEMA and GEL emulsifiers, this shows an opposite trend. The microcapsules produced with GEL exhibited a broad peak at approximately 19.5° and the microcapsules produced with PEMA exhibited a peak at 21.4°. However, the main difference in the peaks lies in the fact that the GEL microcapsules have a much higher CPS value of 191, while the PEMA microcapsules have a lower value, with a peak CPS of 63 only, which is nearly three times lower than the GEL CPS value. It seems that the emulsifiers’ physical presence in the shell may not contribute to the crystalline nature of the overall microcapsule intrinsically, however, the reactions that the emulsifiers promote may be the cause of the variation of the crystalline properties between the PEMA and GEL microcapsules. A potential explanation for this is the nature of the condensation reaction and how the two emulsifiers affect the reaction rate. As previously mentioned, carboxyl groups accelerate the polycondensation reaction between urea and formaldehyde to rapidly creates large-chained thermoset polymers. The carboxyl groups’ reaction rate is accelerated dramatically more than surfactants containing other functional groups such as hydroxyls or amines. In that case, this may lead to more porous and non-compact shells with a more amorphous structure.

Furthermore, the species formed during the polymerization process are also important, especially the ratio between the large chained branched polymers and the methylol urea species. Park and Jeong [36] isolated dimethylol and monomethylol urea and compared the diffractogram with cured pure PUF resin. It was observed that the diffractogram of the monomethylol urea had minimal overlap with the cured UF resin and did not contribute to the overall crystalline nature of the system. Therefore, the carboxyl groups may favour the formation of the monomethylol urea species, which doesn’t contribute to the overall crystallographic nature of the shell material.

The merits of encompassing microcapsules with higher crystallinity include a denser shell structure, in which there is less potential of leakage for the core material, especially in the cases when there are more volatile core materials used. Zhang et al. [19] studied the static leakage of phase change material (PCM) PUF microcapsules. They evaluated that the leakage of the core is ascribed to the permeability of the shell material, in which there is a solution-diffusion mechanism occurring. Subsequently, a more crystalline and dense structure may also lead to higher mechanical integrity, which benefits the long-term storage of the microcapsules.

### 3.7. Self-Healing Microcapsules in Epoxy Primer

**Figure 9:**
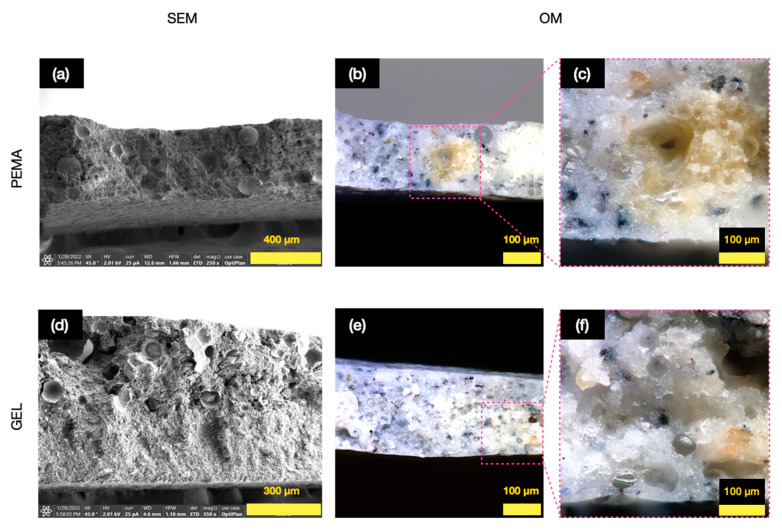
Cross-sectional images of (**a**) SEM image of 5 wt.% PEMA microcapsules primer dispersion, (**b**) OM of the 5 wt.% PEMA microcapsules primer dispersion, (**c**) closer observation of the OM for the 5 wt.% PEMA microcapsules primer dispersion, (**d**) SEM image of 5 wt.% GEL microcapsules primer dispersion, (**e**) OM of the 5 wt.% GEL microcapsules primer dispersion, (**f**) closer observation of the OM for the 5 wt.% GEL microcapsules primer dispersion.

Due to their excellent chemical resistance, anti-corrosion properties and strong paint film adhesion epoxy resins are widely used in coatings [37]. Therefore, 5 wt.% microcapsules were added into a Hempaprime 500 epoxy resin primer to develop a self-healing coating. One important consideration with this coating is the dispersion and the homogeneity of the microcapsules in the dispersion. Therefore, after the addition of the microcapsules into the epoxy primer, a thin layer was snapped, and observed under the SEM and OM conditions, as shown in Figure 9. The cross-sectional images as obtained from the SEM and OM provided clear images of the embedded microcapsules in the primer. For the microcapsules formulated with PEMA, it can be seen on Figure 9a that there are indeed microcapsules embedded in the primer, with spaces where the layer has been snapped off, and some pristine microcapsules still evident there. There seems to be good compatibility with the primer, with no spaces between the capsule shell and the primer material. Furthermore, as observed in Figure 9b, the OM images also show the existence of surviving microcapsules, as well as the release of tung oil when the primer layer was snapped, as seen more clearly in the magnified Figure 9c. For the GEL samples, a similar result was observed. The cross-sectional SEM image of the primer on Figure 9d conveys several microcapsules embedded in the primer. The OM images in Figure 9e,f show the embedded microcapsules, as well as some released tung oil during the breakage.

Boumezgane et al. [38] carried out an experiment similar to this, in which they created self-healing epoxy coatings with microencapsulated polydimethylsiloxane oligomers for corrosion protection applications. It was seen that the microcapsules embedded in their epoxy matrix did not seem to be in contact with each other, however the dispersion was not completely homogeneous. Nonetheless, there was not a clustering effect observed. Similarly, Neto et al. [39] also formulated tung oil microcapsules with a one-component alkyd coating, and their results conveyed the presence of microcapsules with relatively uniform size distributions, with the microcapsules maintaining their shape and morphology. Lang and Zhou [40] also obtained similar results with linseed oil.

Subsequently, the adhesion properties of the primer coating with and without the addition of microcapsules was studied. These tests were carried out in triplicate, and the results can be seen in Table 2. The force required to pull off the coating layer for the pure epoxy primer sample was 12.76 MPa, with a time of 13.8 s. With the addition of both 5 wt.% and 10 wt.% PEMA, this reduced the value to 12.36 and 12.19 MPa, respectively. However, this was not a significant amount, and it is expected that some coating adhesion may be sacrificed with the addition of the microcapsules. The GEL samples also slightly reduced the required force, with a value of 12.66 MPa and 12.27 MPa for the 5 wt.% and the 10 wt.%, respectively. Ultimately, there was not a substantial negative impact on the adhesive strength with the addition of both types of microcapsules to the epoxy primer, and this was con-firmed by Tukey’s HSD analysis, in which the variance in the mean values was insignificant, as seen in Table 3. A similar trend was observed when Samadzadeh et al. [41] carried out pull-off tests for time oil microcapsules embedded in epoxy resins.

### 3.8. The Release of Tung Oil with GEL Microcapsules

As these microcapsules are aimed to be implemented in self-healing applications, such as for corrosion and scratch protection in the marine industry, it is important to observe the rupturing of the microcapsules to observe the release of the encapsulated tung oil. For example, self-healing microcapsules are often entrapped in an epoxy matrix and layered on metallic substrates, such as steel. When the microcapsules containing the self-healing material are exposed to external stimuli, such as external pressure, force, or temperature, the shell material ruptures to release the active self-healing agent. Therefore, to demonstrate the successful encapsulation and the release of the tung oil, the microcapsules formulated with the GEL emulsifier were selected.

**Figure 10:**
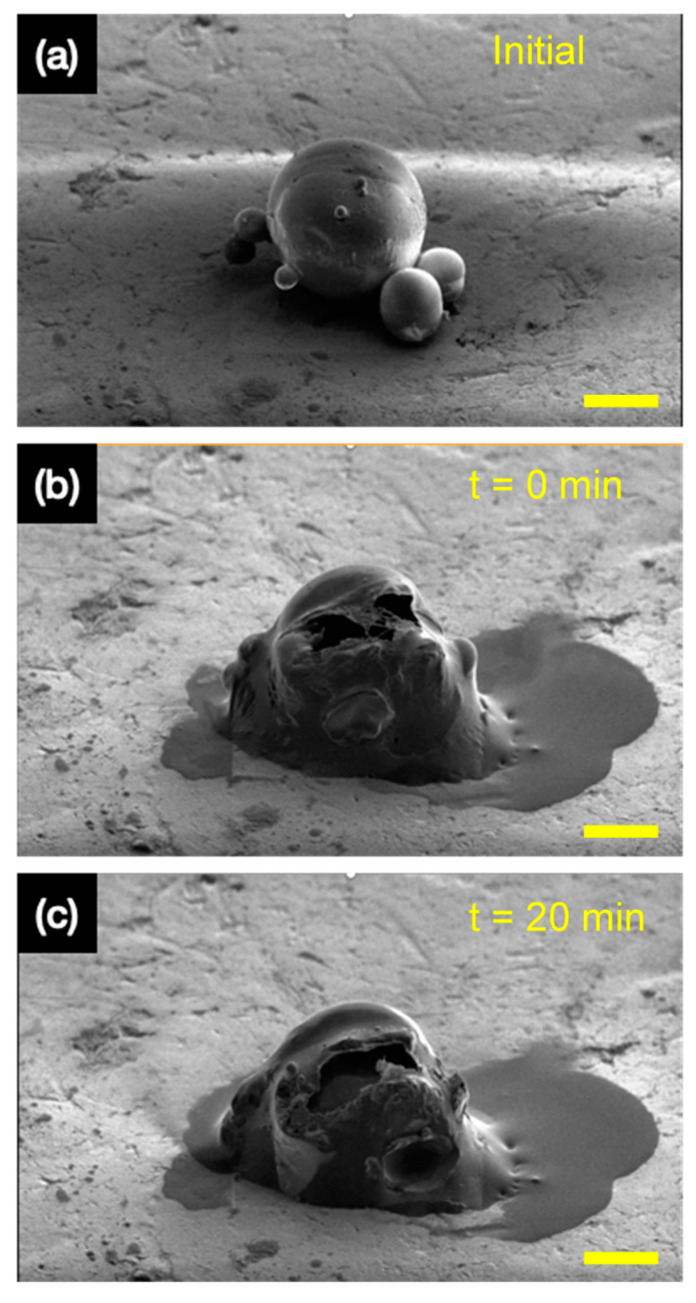
The release of tung oil during the cutting process of the microcapsule with the use of FIB (**a**) microcapsule before the rupture, (**b**) microcapsule after the initial rupture, (**c**) microcapsule 20 min after the initial rupture. All scale bars are 20 µm.

Microcapsule samples were isolated, and then the focussed ion beam (FIB) was used to mill the microcapsule. We propose this completely novel and contemporary method to study the release effect of microcapsules, in which individual microcapsules can be isolated to study the release effect of the active self-healing core more accurately. As shown in Figure 10, this occurred in 3 stages. The uncut microcapsule in Figure 10a was isolated, and then the initial milling proceeded thereafter, which can be seen in Figure 10b. This led to the rapid release of the tung oil, with further release after 20 min observed in Figure 10c. This in situ microscale experiment demonstrates and verifies the successful containment of tung oil and the release behaviour when exposed to external stimuli. With this, the application of the microcapsules produced with the GEL emulsifier can be proposed to be used as a self-healing corrosion protection agent. Although in real life applications the exact source of the external stimuli may be different in terms of force and nature, but nonetheless, it is interesting to observe the release behaviour of individually isolated microcapsules upon rupturing.

### 3.9. Self-Healing Performance in Epoxy Resin

**Figure 11:**
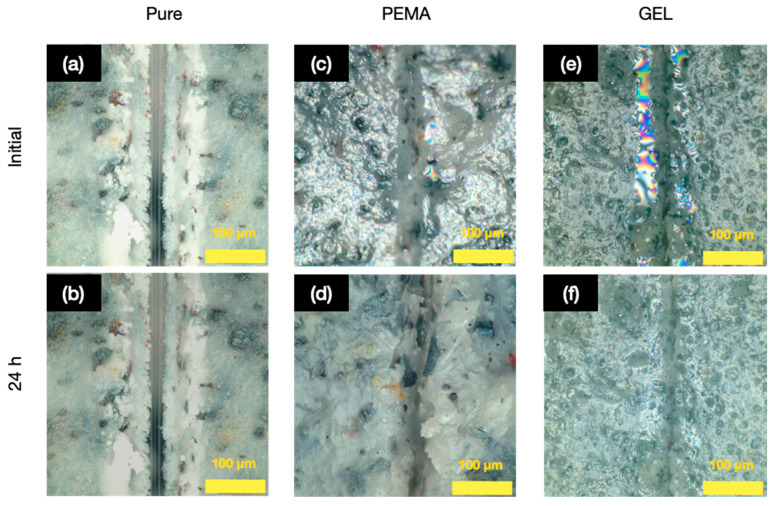
(**a**) Initial scratch for the pure epoxy primer, (**b**) 24 h after scratching the pure epoxy primer, (**c**) initial scratch for the 5 wt.% PEMA embedded epoxy primer, (**d**) 24 h after scratching the 5 wt.% PEMA embedded epoxy primer, (**e**) initial scratch for the 5 wt.% GEL embedded primer, (**f**) 24 h after scratching the the 5 wt.% GEL embedded primer.

As the GEL microcapsules are seen to release the tung oil during the FIB milling method, the microcapsules were then embedded into epoxy primer, and applied onto a small glass slide for observation under SEM and OM, to observe the self-healing phenomena after a scratch. Three samples were created, including the pure epoxy primer with no microcapsules, and one sample with 5 wt.% PEMA microcapsules in the primer, and another sample with 5 wt.% GEL microcapsules in the primer. Figure 11 shows the samples immediately after the scratch, and 24 h after the scratch. As shown in Figure 11a,b, the pure epoxy sample did not exhibit any healing phenomena, with no noticeable reduction in the scratch width after the 24 h elapsed. However, with the 5 wt.% PEMA sample, there seemed to be a healing phenomenon, as well as some leaking of oil, and after the 24 h elapsed, there was a reduction in the gap. As the samples with the microcapsules get ruptured, there is release of tung oil via a capillary action, therefore generating a new film in the scratched area. However, for the samples without the tung oil microcapsules, this process does not occur due to the absence of a healing agent in the epoxy. Furthermore, the GEL samples exhibited large amounts of oil release after the original scratch, as shown by the OM in DIC conditions. This again confirms the storage and release of the tung oil, agreeing with the FIB experiments. Furthermore, the large amounts of tung oil is in agreement with the very high payload values (96.5%) exhibited by the GEL samples. This again signifies the advantage of utilising the GEL emulsifier over the synthetic PEMA alternative, and more effective healing is observed within the same time frame.

**Figure 12:**
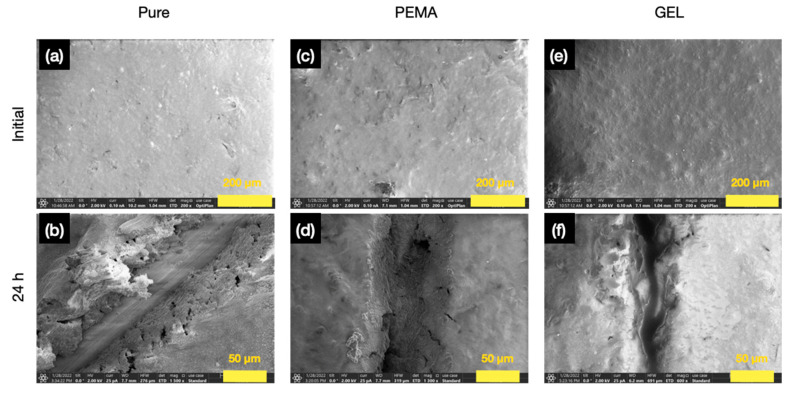
SEM micrographs fo r(**a**) pristine epoxy primer, (**b**) 24 h after scratching the pure epoxy primer, (**c**) pristine 5 wt.% PEMA embedded epoxy primer, (**d**) 24 h after scratching the 5 wt.% PEMA embedded epoxy primer, (**e**) pristine 5 wt.% GEL embedded primer, (**f**) 24 h after scratching the the 5 wt.% GEL embedded primer.

The SEM images in Figure 12 also indicates the significant release of tung oil with the GEL samples, clearly displaying more successful curing of the polymerizing agent. Furthermore, the surface morphology or roughness of the primer and the polymerizing agent is not significantly increased with the addition of the microcapsules in the epoxy primer. As shown previously, the average size of the PEMA microcapsules is ~63 µm, and the GEL microcapsules ~56 µm, and the primer specification thickness is ~162 µm.

**Figure 13:**
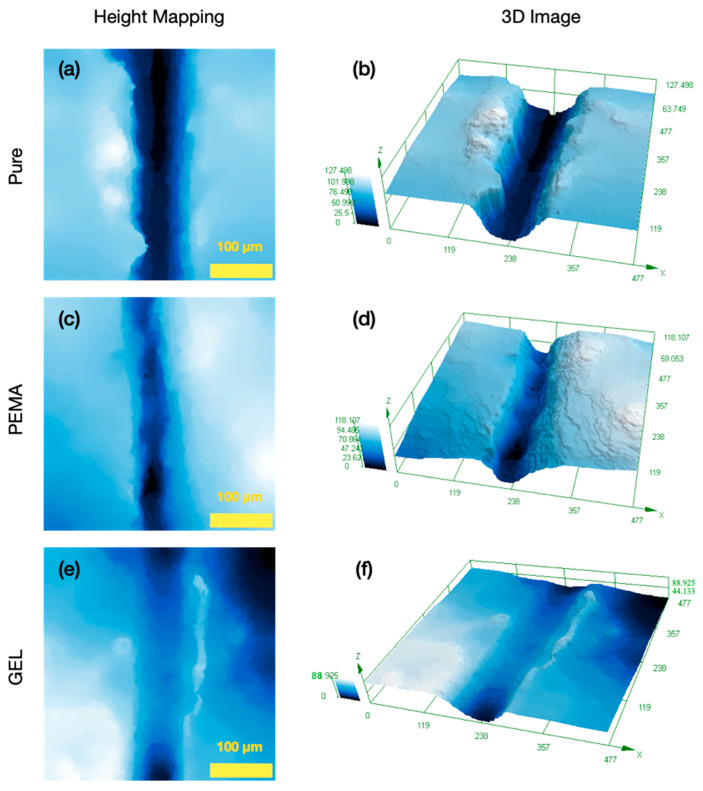
OM imaging of the samples 24-h after the initial scratch; (**a**) pure epoxy primer height mapping, (**b**) pure epoxy primer 3D imaging, (**c**) PEMA microcapsule primer coating height mapping, (**d**) PEMA microcapsule primer coating 3D imaging, (**e**) GEL microcapsule primer coating height mapping, (**f**) GEL microcapsule primer coating 3D imaging. (3D Imaging scale bar in µm).

Height mapping analysis was carried out for quantitative analysis of the self-healing mechanism for the samples with and without the microcapsules embedded in the epoxy primer. The samples were cut, and then observed under the OM after the 24-h curing process. A 3D model was then obtained, in which there was also mapping of the contours. This can be seen in Figure 13. The contour mapping in Figure 13a displays the pure resin 24 h after the scratch, in which the darker regions convey deeper contours, while the lighter blue colour signifies more shallow profiles. Figure 13b exhibits the 3D version of this image, in which a deep scratch is observed after the 24-h period elapsed. For this sample, there was an average scratch depth of 82.64 µm and 81.22 µm before and after the 24-h period, respectively. This can be seen in Table 4. 20 measurements were taken across the profile of the scratch, with an average value obtained. There is a consistent scratch depth through the sample, which is expected, as negligible healing took place for this sample. However, with the PEMA samples, there is seen to be a difference with the contour profile. Table 5 and Table 6 convey that for the initial scratch, there was significant differences in the mean values for the scratch depth, as well as after the 24-h time period elapsed. After this 24-h period, all the samples had significant differences in terms of mean values for scratch depth, which was expected due to the large difference in the final scratch depth value and the healing processes exhibited especially by the GEL sample.

In Figure 13c, there are less darker regions in the contour mapping compared to the pure sample. Furthermore, the initial average scratch depth after the initial scratch was 78.4 µm, and after the 24 h elapsed, this value reduced to 28.72 µm, conveying healing of the scratch with a depth difference of 49.58 µm. This indicates a 63% healing of the material. This is proposed to be a result of the release of the tung oil covering the ruptured area. Additionally, the GEL samples, as seen in Figure 13e expresses even fewer areas of depth after the 24-h elapsed. The 3D image conveyed Figure 13f suggests a much lower scratch depth when compared to the pure epoxy, and the PEMA samples. The initial scratch depth for the GEL samples was 67.68 µm, and after the 24-h, this value reduced to 5.8 µm, displaying a far superior healing with a healing depth difference of 61.88 µm as compared to the PEMA sample. The result implies a 91% healing effect. Overall, the GEL samples exhibited greater results (increment of 28%) when compared to the PEMA samples, and exhibited much more improved healing, again showing the beneficial advantages of using the GEL as the emulsifier in the formulation process.

### 3.10. Corrosion Resistance Performance of the Self-Healing Coating

**Figure 14:**
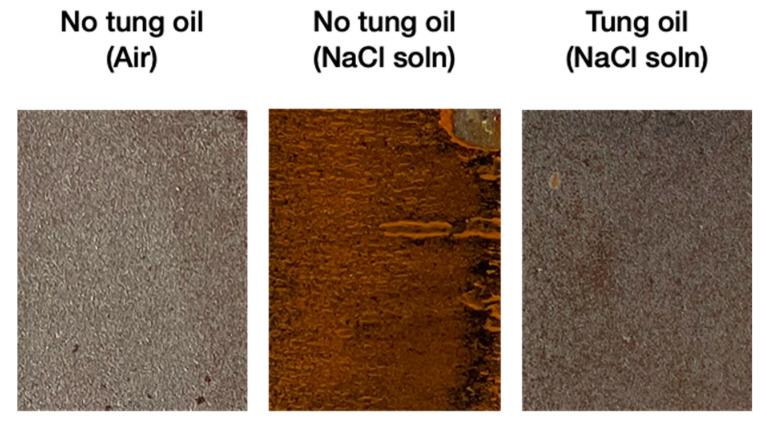
Steel plateswith no tung oil left in air for 24 h, and steel plates with and without tung oil immersed in 3.5 wt.% NaCl solution for 24 h.

As these microcapsules containing the SH tung oil component are to be used for corrosion protection applications, it is pivotal to investigate the effectiveness of the coatings when exerted in corrosive environments. Three sets of steel plates were prepared, one with no addition of tung oil kept in atmospheric conditions, another with no tung oil and immersed in 3.5 wt.% NaCl solution, and one with tung oil also immersed in the NaCl solution. As shown by Figure 14, the tung oil indeed has a large effect in protecting the steel from corrosion, and thus is a suitable core material for corrosion applications.

**Figure 15:**
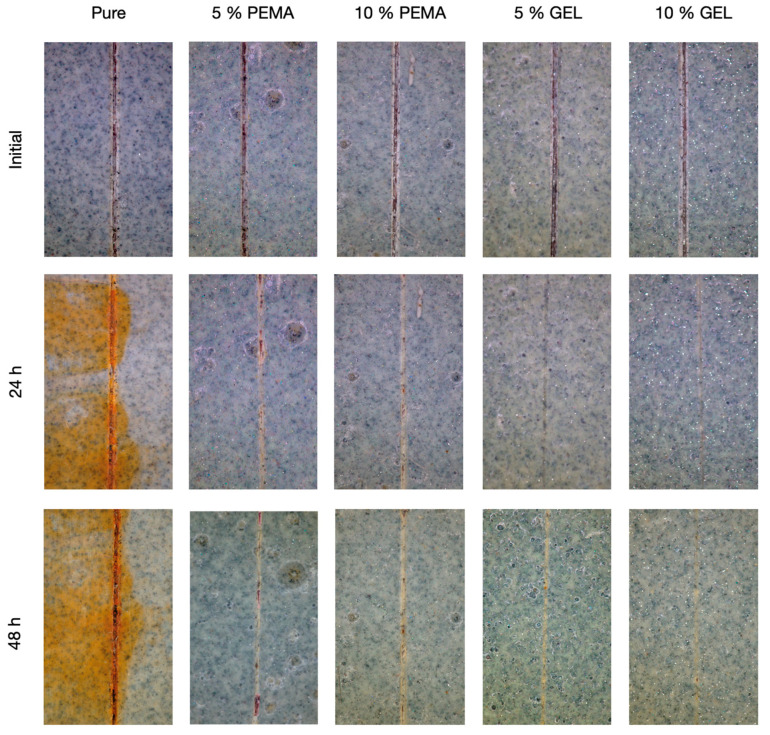
Steel substrates coated with the epoxy primer with and without the addition of 5 wt.% and 10 wt.% PEMA and GEL samples, immersed in 3.5 wt.% NaCl solution for 24 h and 48 h.

The microcapsule-coating mix and the pure coating were then applied onto the steel substrates. For the microcapsule primer mix, there were 5 wt.% and 10 wt.% microcapsule contents prepared. After the application of the coating, a scratch was applied onto the plates, and then the samples were placed in the 3.5 wt.% NaCl solution for various time increments. These results can be seen in Figure 15. Immediately, it is evident that the samples with no microcapsules had significant corrosion after 24 h, with increased corrosion after the 48 h elapsed, due to the lack of protection from the raw steel with the salt solution. Regarding the 5 wt.% and the 10 wt.% PEMA samples, relatively similar results were observed, with minimal corrosion present in the scratch. However, with the 5 wt.% and 10 wt.% GEL samples, the results were the most advantageous, with clear healing of the original scratch, and no visible corrosion after 48 h. This again conveys the advantageous properties of utilising the natural GEL emulsifier over the synthetic PEMA polymer.

## 4. Conclusions

In this work, tung oil was encapsulated with a urea-formaldehyde shell via the use of one-step in situ polymerization. We proposed that the main differences in the morphological properties of the microcapsules stem in the differences in the functional groups of the emulsifiers used in this process. Under such a premise, the conventionally used PEMA emulsifier was compared with the natural GEL emulsifier. The results conveyed GEL as a promising naturally abundant alternative. The microcapsules produced with GEL produced superior payload, yield, and encapsulation efficiency with 96.5, 28.9 and 61.7%, respectively, while PEMA resulted in values of 90.8, 28.6 and 52.6%, respectively.

Furthermore, the GEL microcapsules had a more uniform morphology and a much smoother surface texture comparing with PEMA. As observed in the OM and SEM images, there were very few surface polymers on the surface of the prepared GEL microcapsules. The particle size monitoring during the reaction process also conveyed the differences in the morphological behaviours during the synthesis, in which the PEMA samples produced larger and more broad particle sizes.

Additionally, the GEL samples had a thinner shell by 65% compared to the PEMA samples. The GEL samples also exhibited a more crystalline structure, which alludes to higher hydrolytic stability, which is excellent for long term storage and reduced chances of formaldehyde emission from the shell, which is another added merit in terms of safety and environmental concerns.

Moreover, the FIB process’s novel and innovative milling procedure conveyed the successful entrapment and release of the tung oil upon rupture in the GEL samples. The self-healing was also evaluated for the substances, conveying that the microcapsules containing GEL exhibited a higher healing efficiency of 91%, compared to the 63% healing efficiency established by the PEMA samples. Furthermore, all the samples containing the microcapsules in the epoxy primer coated on the steel substrate established corrosion resistance after 48 h, with the GEL samples conveying exceptional results.

The significance of our findings lies in the fact that the emulsifier of choice can significantly affect the microcapsules’ morphological, crystalline and barrier properties. With this, factors such as the microcapsule size, shell thickness and surface roughness may be fine-tuned and controlled for the intended application by altering the emulsifier of choice.

## Figures and Tables

**Table 1:** Tukey’s statistical analysis for the measurement pairs, for the PEMA and GEL PL, YLD and EE respectfully.

Pair	Tukey’s HSD Q Statistic	Tukey’s HSD *p*-Value	Tukey’s HSD Inference
PEMA PL v. GEL PL	18.0	1.00 × 10^−3^	*p* < 0.05
PEMA YLD v. GEL YLD	2.20	0.20	Insignificant
PEMA EE v. GEL EE	11.90	1.10 × 10^−3^	*p* < 0.05

**Table 2:** Initial scratch depth, 24-h scratch depth and the healing depth differences for the pure epoxy primer, the PEMA microcapsule epoxy primer mix, and the GEL microcapsule epoxy primer mix.

	Adhesion Strength (MPa)	Time until Failure (s)
Pure	12.76 ± 0.3	13.8 ± 0.2
5 wt.% PEMA	12.36 ± 0.2	14.0 ± 0.6
10 wt.% PEMA	12.19 ± 0.1	13.5 ± 0.3
5 wt.% GEL	12.66 ± 0.3	13.9 ± 0.4
10 wt.% GEL	12.27 ± 0.5	13.1 ± 0.5

**Table 3:** Tukey’s statistical analysis for the measurement pairs, for the Pure, 5 wt.% PEMA, 10 wt.% PEMA and 5 wt.% GEL and 10 wt.% GEL PL, adhesion strengths, respectively.

Pair	Tukey’s HSD Q Statistic	Tukey’s HSD *p*-Value	Tukey’s HSD Inference
Pure v. 5 wt.% PEMA	2.35	0.49	Insignificant
Pure v.10 wt.% PEMA	3.35	0.20	Insignificant
Pure v. 5 wt.% GEL	0.59	0.90	Insignificant
Pure v. 10 wt.% GEL	2.68	0.38	Insignificant
5 wt.% PEMA v. 10 wt.% PEMA	1.00	0.90	Insignificant
5 wt.% PEMA v. 5 wt.% GEL	1.76	0.71	Insignificant
5 wt.% PEMA v. 10 wt.% GEL	0.33	0.90	Insignificant
10 wt.% PEMA v. 5 wt.% GEL	2.77	0.35	Insignificant
10 wt.% PEMA v. 10 wt.% GEL	0.67	0.89	Insignificant
5 wt.% GEL v. 10 wt.% GEL	2.10	0.57	Insignificant

**Table 4:** Initial scratch depth, 24-h scratch depth and the healing depth differences for the pure epoxy primer, the PEMA microcapsule epoxy primer mix, and the GEL microcapsule epoxy primer mix.

	Initial Scratch Depth (µm)	24 h Scratch Depth (µm)	Healing Depth Difference (µm)
Pure	82.64 ± 6.2	81.22 ± 4.1	1.42
PEMA	78.4 ± 5.9	28.72 ± 5.3	49.58
GEL	67.68 ± 4.8	5.8 ± 2.6	61.88

**Table 5:** Tukey’s statistical analysis for the measurement pairs, for Pure, PEMA and GEL samples initial scratches.

Pair	Tukey’s HSD Q Statistic	Tukey’s HSD *p*-Value	Tukey’s HSD Inference
Pure v. PEMA	1.30	0.65	Insignificant
Pure v. GEL	4.57	0.04	*p* < 0.05
PEMA v. GEL	3.28	0.13	Insignificant

**Table 6:** Tukey’s statistical analysis for the measurement pairs, for Pure, PEMA and GEL samples 24 h after initial scratches.

Pair	Tukey’s HSD Q Statistic	Tukey’s HSD *p*-Value	Tukey’s HSD Inference
Pure v. PEMA	21.50	1.01 × 10^−3^	*p* < 0.05
Pure v. GEL	30.90	1.01 × 10^−3^	*p* < 0.05
PEMA v. GEL	9.37	1.37 × 10^−3^	*p* < 0.05

## Data Availability

All data is contained within this article.
